# Cigarette Smoking, Risky Alcohol Consumption, and Marijuana Smoking among University Students in Germany: Identification of Potential Sociodemographic and Study-Related Risk Groups and Predictors of Consumption

**DOI:** 10.3390/healthcare11243182

**Published:** 2023-12-16

**Authors:** Thilo A. Hoff, Sebastian Heller, Jennifer L. Reichel, Antonia M. Werner, Markus Schäfer, Ana Nanette Tibubos, Perikles Simon, Manfred E. Beutel, Stephan Letzel, Thomas Rigotti, Pavel Dietz

**Affiliations:** 1 Institute of Occupational, Social and Environmental Medicine, University Medical Centre of the Johannes Gutenberg University, 55131 Mainz, Germany; thilo.hoff@web.de (T.A.H.); seheller@uni-mainz.de (S.H.); jreichel@uni-mainz.de (J.L.R.); letzel@uni-mainz.de (S.L.); 2 Department of Psychosomatic Medicine and Psychotherapy, University Medical Center of the Johannes Gutenberg University, 55131 Mainz, Germany; antonia.werner@unimedizin-mainz.de (A.M.W.); tibubos@uni-trier.de (A.N.T.); manfred.beutel@unimedizin-mainz.de (M.E.B.); 3 Department of Communication, Johannes Gutenberg University, 55122 Mainz, Germany; markus.schaefer@uni-mainz.de; 4 Nursing Science, Diagnostics in Healthcare and E-Health, Trier University, 54296 Trier, Germany; 5 Department of Sports Medicine, Rehabilitation and Disease Prevention, Institute of Sport Science, Johannes Gutenberg University, 55122 Mainz, Germany; simonpe@uni-mainz.de; 6 Department of Work, Organizational and Business Psychology, Institute of Psychology, Johannes Gutenberg University, 55122 Mainz, Germany; rigotti@uni-mainz.de; 7 Leibniz Institute of Resilience Research, 55122 Mainz, Germany

**Keywords:** substance use, drugs, college, university, tertiary education

## Abstract

(1) Background: Cigarette smoking, risky alcohol consumption, and marijuana smoking are the most common behaviors related to legal and illicit drug use worldwide, including among university students. To plan effective evidence-based programs to prevent the risky consumption of these substances among university students, the present study aimed to identify potential sociodemographic and study-related risk groups and predictors of consumption. (2) Methods: A cross-sectional online health survey with approximately 270 health-related items was conducted among students at the University of Mainz, Germany. Cigarette smoking, risky alcohol consumption (AUDIT-C score: female ≥ 4, male ≥ 5), and marijuana smoking were chosen as dependent variables. Of the 270 health-related items, 56 were chosen as independent variables and collated into five groups (sociodemographic, psychological, study-related psychosocial, general psychosocial and health behavior). The prevalence of cigarette smoking, risky alcohol consumption, and marijuana smoking was assessed using established and validated instruments. Pearson’s chi-square test was used to analyze the differences in prevalence between the sociodemographic and study-related groups, and binary logistic regression was used for analyses with stepwise inclusion of the five variable groups. (3) Results: Of the 3991 university students who entered the analyses, 14.9% reported smoking cigarettes, 38.6% reported risky alcohol consumption, and 10.9% reported smoking marijuana. The prevalence of these differed between genders, fields of study, and aspired degree level, among other factors. Binary logistic regression analyses revealed nine significant predictors (*p* ≤ 0.05) of cigarette smoking (Nagelkerke R^2^ = 0.314), 18 significant predictors of risky alcohol consumption (Nagelkerke R^2^ = 0.270), and 16 significant predictors of marijuana smoking (Nagelkerke R^2^ = 0.239). (4) Conclusions: This study showed cigarette smoking, risky alcohol consumption, and marijuana smoking among university students in Germany to be associated with multiple factors, especially health behaviors. Furthermore, each of the substances was highly associated with each of the two other substances we examined. Other variable groups, such as psychological or psychosocial variables, seemed to play a rather minor role. Therefore, our recommendation for future prevention programs is that substance use among university students should be addressed as a whole, not just in terms of specific substances.

## 1. Introduction

Cigarette smoking, risky alcohol consumption, and marijuana smoking are known to be the most common behaviors related to legal and illicit drug use worldwide [1]. Globally, 15.2% of all adults are daily smokers [2]. According to the latest wave of the Global Burden of Disease (GBD) study, in 2019, 32.7% of men and 6.6% of women were characterized as current cigarette smokers [3]. In Western Europe, the overall prevalence of current cigarette smoking is quite similar, but the difference between men and women is much smaller. For example, the GBD study reported the prevalence of current cigarette smoking as being 28.8% for men and 22.7% for women in Western Europe, and Lange et al. [4] reported to be 28.7% for men and 19.5% for women in Germany. A particularly high prevalence of current cigarette smoking was reported among people living in Greece (32.2% women, 44.1% men), France (31.3% women, 36.9% men), and Austria (26.1% women, 36.1% men) [3]. In Germany, cigarette smoking still accounts for the majority of tobacco consumption [5,6,7]. Similar to the international figures presented above, recent data revealed that 23.0% of women and 29.9% of men in Germany are current cigarette smokers [3]. Furthermore, according to the 2018 Annual Survey of the Federal Government, 23.3% of all German adults reported smoking in the previous 30 days [5]. A closer look at the age distribution of cigarette smokers in Germany showed in 2017 that younger people smoke at a significantly higher rate than the population as a whole. For example, a study of 24,016 adults from Germany showed that 35.1% of men and 28.4% of women aged 18–29 years were smokers, compared to only 9.2% of men and 6.8% of women aged 64 years and older [8]. In the younger age group, university students are no exception [9]. According to a recent survey among 7394 university students in Germany, 18.5% of the participants reported smoking cigarettes [10]. These figures are very similar to those reported by Velten et al., who surveyed a sample of 2991 university students and identified 20.8% as cigarette smokers [11]. Although the number of smokers declined slightly in the years up to the pandemic, most smoking usually starts in the age range between 15 and 24 years [12].

There are different definitions of risky alcohol consumption [13]. In the US, it is defined as having more than seven alcoholic drinks per week for women (or >3 drinks per occasion) and more than 14 for men (or >4 drinks per occasion) [14]. In German-speaking countries, it is defined by drinking more than 12 g of alcohol per day for women and 24 g of alcohol per day for men [15,16]. Globally, 32.5% of people are classified as current drinkers, defined as having at least one standard drink of 10 g of pure ethanol per day [17]. Prevalence is higher in Western countries. For example, a recent review of 4,152,000 participants reported a prevalence of 30.5% for risky alcohol consumption within the previous year [2]. In Germany, 16% of the general population regularly consume risky amounts of alcohol [4]. University students appear to be a particularly vulnerable population for risky alcohol consumption [18,19]. For example, in the US, Slutske et al. [20] and others identified increased risk specifically in the college student group, compared to non-college students of the same age and the general population [20,21]. Their results support several other international studies conducted within the college and university student populations [19,22,23,24,25]. Looking more closely at German university students, the prevalence of risky alcohol consumption is at a very high level [10,26]. For example, Grützmacher et al. [10] reported a prevalence of 40.6% for risky alcohol consumption in a sample of 6198 university students.

According to the World Drug Report 2021, marijuana is the most commonly used illicit drug in the world, and the global prevalence of its use was estimated to be around 4% in 2019 [27]. Significantly higher rates have been reported for North America and Western Europe. For example, the SAMSHA study, a national survey of drug use and health in the US, showed that 18.0% of 44,958 participants aged 18 years and older had used marijuana within the previous year [28]. In addition, the United Nations has reported that 14.5% of adults in North America and 7.8% in Western Europe consume marijuana [29]. Looking at younger age groups, 15% of Europeans aged 15–34 years reported using cannabis in the previous year. In Germany, the prevalence of use in this age group was quite comparable, at 16.9% [30]. In addition, the 18–24 age group showed an even higher prevalence of use, of 22.0%. Marijuana is the most commonly used drug among university students [31]. Among all graduated US college students, more than 50% have used marijuana at least once in their lifetime, 15.8% within the previous year [28]. In addition, other studies have reported a prevalence of 29.1% in the past year [32]. Among university students in Germany, a lifetime prevalence of marijuana use of 45.6% and a 12-month prevalence of 20.6% were reported. Furthermore, the 12-month prevalence of marijuana use in Germany approximately doubled between 1990 and 2018 [5,33,34], indicating an increase in marijuana consumption in recent decades [10,23,35].

From a public health perspective, the prevalence of cigarette smoking, risky alcohol consumption, and marijuana smoking, especially among university students, is alarming, since the use of these substances is associated with various physiological and psychological side effects, can lead to addiction, and increases mortality. Specifically, cigarette smoking is one of the leading factors for premature mortality and years of life lost due to disease and disability [1,36]. It is associated with an increased risk of coronary heart disease and atherosclerosis [37,38], pulmonary disease such as COPD [39,40], multiple types of cancer [41,42], and many other diseases [1,6,43,44]. There is no risk-free level of exposure to tobacco smoke, and even occasional tobacco smoking (e.g., light smoking) is sufficient to increase mortality and morbidity [45,46]. In 2010, alcohol use was ranked third in the global burden of disease [1]. Risky alcohol consumption can lead to liver cirrhosis or injury [47] and is associated with cardiovascular diseases such as hypertension, hypercholesterolemia, and type 2 diabetes [48]. It is also one of the leading risk factors for cancer [1,36,49,50]. Smoking marijuana can lead to respiratory disease and, especially in adolescents, psychotic disorders [51]. For example, a systematic review of longitudinal studies reported increased rates of mental health problems after marijuana consumption [52]. In addition, according to Manthey et al. [53], smoking marijuana leads to higher risk for psychotic disorders, acute cognitive impairment, traffic injuries, respiratory problems, and poorer pregnancy outcomes [53,54]. Studies suggest that in Germany, marijuana is mostly (87.2%) consumed in the form of cigarettes (also known as “joints”) with tobacco [55]. Therefore, an adjustment for the adverse health effects of tobacco must be considered when studying the smoking of marijuana. In addition, cigarette smoking, risky alcohol consumption, and marijuana smoking can lead to addiction. Withdrawal from cigarette smoking and the nicotine contained in joints for marijuana smoking can lead to, among other symptoms, to frustration, anger, a depressed mood, and insomnia [56]. Alcohol consumption results in the suppression of nervous system excitability and rebounds during withdrawal [47]. It can lead to craving, a depressed mood, increased agitation, and seizures [57].

Another aspect that is discussed in the literature is the gateway theory, or the drug gateway hypothesis [58]. This states that alcohol consumption promotes the use of tobacco and vice versa, and that both drugs promote the use of marijuana. In turn, marijuana is discussed as promoting the use of other illicit drugs, such as opioids and amphetamines [59,60,61,62,63]. Although the different patterns of use are not yet fully understood, a link between these substances is likely. In addition to adverse health effects, cigarette smoking, risky alcohol consumption, and marijuana smoking generate high socioeconomic costs each year [64,65,66,67].

To plan evidence-based and effective programs to prevent cigarette smoking, risky alcohol consumption, and marijuana smoking among university students, it is important to understand the conditions and factors that predict the use of these substances among this target group. Therefore, potential correlates (associated factors) or determinants (factors with a causal relationship) need to be explored for each substance. In this context, several studies have examined the relationship between sociodemographic, study-related, or psychosocial factors and cigarette smoking, risky alcohol consumption, or marijuana smoking [11,20,24,25,26,43,68,69,70,71,72,73,74,75,76,77,78,79,80,81,82]. For example, Skidmore et al. [81] identified study-related variables and specific peer-groups associated with substance use among university students. Van Hooijdonk et al. [82] identified an association between study-related characteristics and smoking, drinking alcohol, and consuming marijuana in the Netherlands. Others have shown associations between psychosocial factors [11,24,25] or health behaviors [26,68] and substance use.

However, to the best of our knowledge, no study has examined sociodemographic, psychological, study-related, and general psychosocial as well as health behavior variables simultaneously in one regression model for each of the investigated substances. By doing so, researchers may be able to paint a more comprehensive picture of the predictors of cigarette smoking, risky alcohol consumption, and marijuana smoking among university students. In summary, empirical studies of cigarette smoking, risky alcohol consumption, and marijuana smoking among university students are heterogeneous in their methodology (e.g., definition of consumption, surveyed groups) and results [11,20,24,25,26,43,68,69,70,71,72,73,74,75,76,77,78,79,80]. Furthermore, there is a significant lack of knowledge about the potential factors that may predict cigarette smoking, risky alcohol consumption, and marijuana smoking among university students. Therefore, the present study aims to address this gap by (i) assessing the prevalence of cigarette smoking, risky alcohol consumption, and marijuana smoking among university students at a large university in Rhineland-Palatinate, Germany; (ii) identifying potential sociodemographic and study-related risk groups for substance use, especially with regard to age, gender, field of study, semester, and aspired degree level; and (iii) examining for the first time in a single regression model the predictors of substance use, including sociodemographic and study-related variables, psychological variables, general and study-related psychological variables, and health behavior, simultaneously. These results may contribute to the discussion on developing and implementing prevention strategies that target cigarette smoking, risky alcohol consumption, and marijuana smoking among university students.

## 2. Materials and Methods

### 2.1. Study Design and Survey Procedure

As part of the Healthy Campus Mainz project, a cross-sectional online health survey was conducted during the 2019 summer term (June and July). All registered students of the University of Mainz (approximately 31,000) were invited to participate via the central mailing list of the university. An email was sent to all students enrolled at the university at that time. The students received this email in their official mailing account, to which the university also sends important information about grades. Monetary and nonmonetary incentives and four reminder emails were used to increase participation. Participants had to be enrolled in at least one subject of study at the university. Answering demographic variables and at least one question on health topics was a prerequisite for inclusion. A full version of the survey (including the variables that were not used in this study) and a detailed reflection of the survey procedure can be found in Reichel et al. [83]. An introduction at the beginning of the online questionnaire briefly explained the background and purpose of the study, which was followed by a statement that participation would be anonymous and voluntary. Informed consent was obtained at the beginning of the survey. Ethical approval to conduct the study was obtained from the Ethics Committee of the Medical Association of Rhineland-Palatinate (No. 2019-14336).

### 2.2. Measures

The online questionnaire covered a wide range of health-related aspects and included approximately 270 items. We used established and validated instruments when available and self-developed scales as little as possible. For the present study, we selected 56 variables as independent variables and summarized them into five groups. Further, we selected three variables (cigarette smoking, alcohol consumption, and marijuana smoking) as dependent variables. A list of all surveyed topics and specific items covered can be found in Reichel et al. [83].

The three dependent variables in the present survey are highlighted here in more detail. To assess the prevalence of cigarette smoking, we asked about the frequency of consumption. The question (translated) was “Do you smoke cigarettes?”. The following answers could be chosen: “never”, “formerly occasionally”, “formerly regularly”, “currently occasionally”, and “currently regularly”. We defined “currently occasionally” and “currently regularly” as “cigarette smoking”. The prevalence of risky alcohol consumption was assessed using the AUDIT-C questionnaire. “AUDIT-C” stands for “Alcohol Use Disorder Identification Test—Consumption”. It is the short version of the original 10-item questionnaire published by the World Health Organization [84] and includes the first three questions of the AUDIT questionnaire. It assesses the frequency of alcohol consumption, the amount of alcohol consumption, and the frequency of excessive alcohol consumption (more than five drinks in one sitting). Scores range from zero to 12. The established cut-off of more than 4 points for women and more than 5 points for men was used to categorize the variable as risky alcohol consumption. The AUDIT-C has been shown in many publications to be a simple instrument for measuring risky alcohol consumption. It can be used to select individuals at high risk for alcohol use disorder or dependence [85,86,87,88,89]. The first question (translated) was: 1. “How often do you have a drink containing alcohol?”. The answer could be one of the following five choices: “never” (0 points), “monthly or less” (1 point), “2–4 times a month” (2 points), “2–3 times a week” (3 points), or “4 or more times a week” (4 points). The second question was “How many standard drinks containing alcohol do you have in a typical day?”. The answer could be one of the following five choices: “1 or 2” (0 points), “3 or 4” (1 point), “5 or 6” (2 points), “7 to 9” (3 points), or “10 or more” (4 points). The third question was: “How often do you have six or more drinks on one occasion?”. As response options, one of the following five items could be selected: “Never” (0 points), “less than monthly” (1 point), “monthly” (2 points), “weekly” (3 points), or “daily or almost daily” (4 points). To assess the prevalence of marijuana smoking, we evaluated the frequency of smoking. The question (translated) was “Do you smoke marijuana?”. The following answers could be chosen: “never”, “formerly occasionally”, “formerly regularly”, “currently occasionally” or “currently regularly”. We defined “currently occasionally” and “currently regularly” as “marijuana smoking”.

To predict cigarette smoking, risky alcohol consumption, and marijuana smoking, 56 independent variables related to the research questions were selected from the questionnaire. A list of the specific variables, scales, and items used in the present study, as well as their references and specific questions with response options (for self-constructed items), is provided in Supplementary Table S1. These 56 variables were classified into five different groups according to the factor groups of the current research, as described in the introduction [90]. These were sociodemographic variables (16 variables, e.g., gender, age, semester, field of study), psychological variables (six variables, e.g., depressive symptoms, loneliness), study-related psychosocial variables (18 variables, e.g., social support from fellow students, competence for self-motivation), general psychosocial variables (five variables, e.g., self-criticism, impulsiveness), and health behavior variables (11 variables, e.g., healthy diet, fruit and vegetable consumption, physical activity, use of neuroenhancement). Regarding the independent variable “use of pharmacological neuroenhancement” (including substances such as methylphenidate, amphetamines, atomoxetine, modafinil, ecstasy, ephedrine, marijuana, cocaine, or crystal meth), an analysis of Heller et al. (2022) [90], from our dataset, showed marijuana to be the most commonly used pharmacological neuroenhancer. Therefore, in the present study, we excluded marijuana as a form of pharmacological neuroenhancement to avoid confounding with the dependent variable marijuana smoking. The term “pharmacological neuroenhancement” (PN) is generally defined as the use of illicit or prescription drugs by healthy individuals for cognitive-enhancing purposes [91,92], such as enhancing alertness, attention, concentration, memory, or mood [93,94]. According to this definition, the so-called soft neuroenhancers (e.g., energy drinks, caffeine tablets) were not included. There are many inconsistencies and differences in the definition of neuroenhancement [95,96], but a full discussion of these would go beyond the scope of this research. 

### 2.3. Data Analysis

Descriptive statistics are presented as means with standard deviations (SD) for continuous scaled variables and as percentages and numbers for noncontinuous scaled variables. To analyze differences in prevalence between sociodemographic and study-related groups, contingency analyses of categorical variables were performed using Pearson’s chi-square (χ^2^) test. Multicollinearity of the 56 independent variables (Supplementary Table S1) was assessed using a collinearity matrix and the variance inflation factor. Correlations greater than 0.75 were excluded. In the next step, pretests were performed to assess the association of each independent variable with the three dependent variables. The dependent variables cigarette smoking, risky alcohol consumption, and marijuana smoking were dichotomized (yes/no). ANOVA was used for linear scaled variables, Pearson’s chi-square test was used for ordinal scaled variables, and Cramer’s V-test was used for nominal scaled variables (Supplementary Tables S2–S4). To predict the three dependent variables, cigarette smoking, risky alcohol consumption, and marijuana smoking, all variables with a significant association (*p* ≤ 0.001) in the pretest were included in a binary logistic regression with stepwise inclusion of the five variable groups. In the binary logistic regression models, variables with significant associations at a level of *p* ≤ 0.05 were classified as possible predictors. Nagelkerke’s R^2^ was calculated to check the strength of each regression model. Data were analyzed using IBM SPSS 23.

## 3. Results

A total of 4351 university students participated in the survey, of whom *N* = 3991 answered the questions with regard to the dependent variables (alcohol consumption, cigarette smoking, and marijuana smoking) and were included in the analyses. The mean age of the sample was 23.8 (±4.3) years, and 71.4% (*n* = 2848) of participants were female. The mean semester was 7.2 (±4.8), and 16.8% (*n* = 651) of the students reported being in their first year (first or second semester). Regarding the degree level being aspired to, 52.3% (*n* = 2088) were aiming for a bachelor’s degree, 21.2% (*n* = 847) were aiming for a master’s degree, 22% (*n* = 878) were aiming for a state examination (a special program that is specific to German students studying, e.g., law or medicine), and 3.5% (*n* = 139) were aiming for a doctoral degree. Regarding the field of study, 18.1% (*n* = 720) indicated STEM (science, technology, engineering, and mathematics); 18.1% (*n* = 719) indicated social science, media, and sports; 20.2% (*n* = 804) indicated linguistics, humanities, and cultural science; 13.3% (*n* = 530) indicated medicine; 12.9% (*n* = 512) indicated law and economics; and 15.5% (*n* = 616) indicated education (aspiring teachers). All of the participant’s sociodemographic and study-related characteristics of the participants are presented in Table 1.

### 3.1. Prevalence, Risk Groups, and Predictors of Cigarette Smoking

As shown in Table 2, 14.9% (*n* = 592) of all students smoked cigarettes. Regarding potential risk groups for cigarette smoking, women (13.4%) smoked significantly (*p* ≤ 0.001) less than men (18.0%) or diverse (34.4%) participants. Concerning the field of study, we assessed a significantly (*p* ≤ 0.001) lower prevalence in students from the field of education (9.8%) compared those from the fields of social science, media, and sports (16.7%); linguistics, humanities, and cultural sciences (18.3%); and law and economics (16.4%). Furthermore, the prevalence differed significantly (*p* ≤ 0.001) between students of medicine (11.2%) and of linguistics, humanities, and cultural sciences (18.3%).

**Figure 1:**
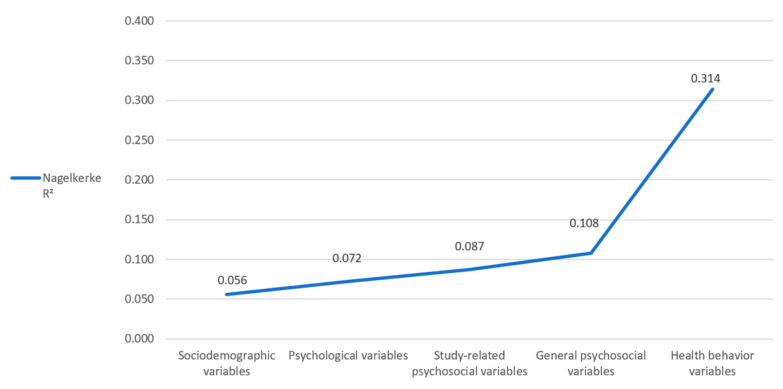
Regression model for cigarette smoking. Changes in Nagelkerke R^2^ by stepwise inclusion of the different variable groups.

Of the 56 independent variables that were previously selected for this study, 24 were significantly associated with cigarette smoking in the pretests, and these were included in the binary logistic regression analysis (Supplementary Table S2). Binary logistic regression revealed nine significant predictors (*p* ≤ 0.05), including 3448 (86.4%) students, for the final regression (Table 3). Negatively related variables were migrant background (OR = 0.585), use of soft neuroenhancement within the past 12 months (OR = 0.593), and former occasional marijuana smoking (OR = 0.790). Positively associated variables were impulsiveness (OR = 1.106), general anxiety (OR = 1.114), risky alcohol consumption (OR = 1.336), current occasional marijuana smoking (OR = 1.488), first study (OR = 2.413), and current regular marijuana smoking (OR = 2.971). The Hosmer–Lemeshow goodness of fit test revealed a chi-square of 2.835, with a significance of 0.944. The stepwise inclusion of the five variable groups revealed a Nagelkerke R^2^ of 0.056 after inclusion of the sociodemographic variables, 0.072 (+0.016) after inclusion of the psychological variables, 0.087 (+0.015) after inclusion of the study-related psychosocial variables, 0.108 (+0.021) after inclusion of the general psychosocial variables, and 0.314 (+0.206) after inclusion of the health behavior variables (Figure 1). Thus, our final model explains 31.4% of the variance in cigarette smoking.

### 3.2. Prevalence, Risk Groups, and Predictors of Risky Alcohol Consumption

In our sample, 38.6% (*n* = 1537) of all students demonstrated a risky alcohol consumption pattern. With regard to potential sociodemographic and the study-related risk groups (Table 4), students younger than or equal to 23 years of age had a significantly higher prevalence of risky alcohol consumption (40.8%, *p* = 0.001) than those aged 24 years and older (35.7%). Furthermore, the prevalence was significantly higher among first year students (42.3%, *p* = 0.030) compared to students in later years (37.8%). Regarding the field of study, the prevalence was significantly higher (*p* = 0.001) among students in the fields of social science, media, and sport (43.2%) and law and economics (43.0%) compared to those in linguistics, humanities, and cultural science (33.5%).

Of the 56 selected independent variables, 24 were significantly associated with risky alcohol consumption in the pretest, and these were included in the binary logistic regression analysis (Supplementary Table S3).

The binary logistic regression analysis revealed 18 significant predictors (*p* ≤ 0.05), including 2908 (72.9%) students, for the final regression (Table 5). The negatively related variables were use of pharmacological neuroenhancement within the past 12 months (OR = 0.625), first study (OR = 0.693), relationship status (joint household) (OR = 0.736), first study (no, graduated before) (OR = 0.745), use of soft neuroenhancement within the past 12 months (OR = 0.799), fruit consumption per day (OR = 0.822), and loneliness (OR = 0.931). Positive associations were detected for procrastination (OR = 1.017), impulsiveness (OR = 1.100), social support by fellow students (OR = 1.205), physical activity (active, beneficial to health) (OR = 1.303), social media use (OR = 1.323), employment (yes, marginally employed) (OR = 1.381), currently regularly cigarette smoking (OR = 1.426), migrant background (OR = 1.523), part-time employment (OR = 1.527), currently occasionally marijuana smoking (OR = 1.600), and currently occasionally cigarette smoking (OR = 1.691). The Hosmer–Lemeshow goodness of fit test revealed a chi-square of 7.430 with a significance of 0.491.

**Figure 2:**
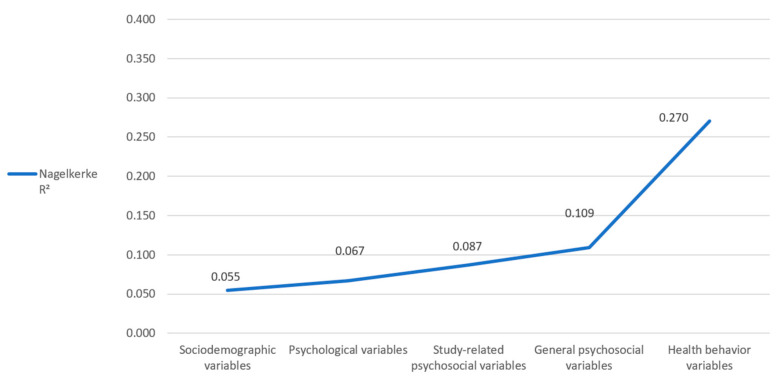
Regression model for alcohol consumption (AUDIT-C score: female ≥ 4, male ≥ 5). Changes in Nagelkerke R^2^ by stepwise inclusion of the different variable groups.

Stepwise inclusion of the five groups of variables revealed a Nagelkerke R^2^ of 0.055 after inclusion of the sociodemographic variables, 0.067 (+0.012) after inclusion of the psychological variables, 0.087 (+0.020) after inclusion of the study-related psychosocial variables, 0.109 (+0.022) after inclusion of the general psychosocial variables, and 0.270 (+0.161) after inclusion of the health behavior variables (Figure 2). Thus, our final model explains 27% of the variance of risky alcohol consumption.

### 3.3. Prevalence, Risk Groups, and Predictors of Marijuana Smoking

In our sample, 10.9% (*n* = 435) of all students smoked marijuana, as defined above (currently occasionally or currently regularly). With regard to potential sociodemographic and study-related risk groups (Table 6), women (9.0%) smoked significantly (*p* ≤ 0.001) less marijuana than men (15.4%) or diverse students (25.0%). Students aged 23 years or younger had a significantly higher prevalence (11.8%, *p* = 0.034) of marijuana smoking compared with those aged 24 years or older (9.7%). With regard to the aspired degree level, bachelor’s students (13.0%) had a significantly higher prevalence (*p* ≤ 0.001) of marijuana smoking than aspirants of state examination (8.7%) or doctoral aspirants (3.6%). Regarding the field of study, we detected a significantly (*p* ≤ 0.001) higher prevalence among students in the field of social science, media, and sports (15.7%) than those in STEM (9.3%), medicine (8.3%), and education (9.1%).

Of the 56 selected independent variables, 16 were significantly associated with marijuana smoking in the pretest, and these were included in the binary logistic regression analysis (Supplementary Table S4), which yielded 12 predictors (*p* ≤ 0.05), including 3435 (86.1%) students, for the final regression (Table 7). Negatively related variables were aspired degree level (doctoral degree) (OR = 0.169), pharmacological neuroenhancement within the past 12 months (OR = 0.646), soft neuroenhancement within the past 12 months (OR = 0.691), self-endangering behavior (OR = 0.827), and semester hours per week (OR = 0.977).

**Figure 3:**
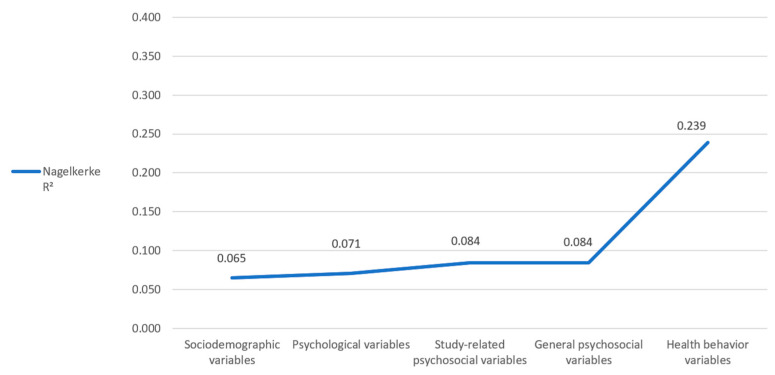
Regression model for marijuana smoking. Changes in Nagelkerke R^2^ by stepwise inclusion of the different variable groups.

Positive associations were detected with risky alcohol consumption (OR = 1.209), gender (male) (OR = 1.314), first study (OR = 1.398), physical activity (moderately active) (OR = 1.451), physical activity (active, beneficial to health) (OR = 1.641), currently regularly cigarette smoking (OR = 1.959), and currently occasionally cigarette smoking (OR = 2.007). The Hosmer–Lemeshow goodness of fit test revealed a chi-square of 11.281, with a significance of 0.186. Stepwise inclusion of the five groups of variables revealed a Nagelkerke R^2^ of 0.065 after inclusion of the sociodemographic variables, 0.071 (+0.006) after inclusion of the psychological variables, 0.084 (+0.013) after inclusion of the study-related psychosocial variables and general psychosocial variables, and 0.239 (+0.15.1) after inclusion of the health behavior variables (Figure 3). Thus, our final model explains 23.9% of the variance in risky alcohol consumption.

## 4. Discussion

The aims of the present study were to (i) assess the prevalence of cigarette smoking, risky alcohol consumption, and marijuana smoking among university students at a large university in Rhineland-Palatinate, Germany; (ii) identify potential sociodemographic and study-related risk groups for substance use, especially with regard to age, gender, field of study, semester, and aspired degree level; and (iii) examine predictors of substance consumption, including sociodemographic and study-related variables, psychological variables, general and study-related psychosocial variables, and health behavior variables in a regression model. The results show that models with groups of sociodemographic and study related variables, psychological variables, general and study related psychosocial variables, and health behavior related variables as predictors are suitable for explaining the prevalence of cigarette smoking, risky alcohol consumption, and smoking marijuana among university students. In each of these three models, the group of health behavior variables specifically showed the highest impact on consumption patterns. In particular, the consumption of each substance studied was predicted by the consumption of each of the other substances studied. With regard to the first aim, our results are in line with the current literature, which reports particularly high prevalence rates for the three types of substance use [20].

### 4.1. Cigarette Smoking

With regard to cigarette smoking, we detected significantly more users in the group of male and diverse students compared to female students, and significantly fewer cigarette smokers in the group of aspiring teachers compared to other fields of study. In particular, the gender difference was observed to be consistent with global trends [97] and previous studies from Germany [5]. One reason for this may be that women are more engaged in seeking health information and take fewer risks than their male peers [98]. Regarding the field of study, there are no recent data available comparing different groups of students in Germany with regard to their smoking behavior.

Among the sociodemographic variables, migrant background and having dropped out of studies in the past emerged as strong predictors of cigarette smoking. Interestingly, migrant background was negatively associated with cigarette smoking in our sample, which is contrary to previous studies [73,99]. One possible reason for this could be an overrepresentation of female participants in the subgroup of students with an immigrant background, with the consequence that the female characteristic is more likely to be represented here than the specified migrant background. Another reason could be the ”healthy migrant” paradox, which describes that people with migrant background have better health behaviors than natives [100] and, in particular, are less likely to smoke cigarettes [100,101]. This effect is particularly evident in the first few years after migration—for example, young people who leave their home country to study abroad. As the length of stay increases, health behaviors become more similar to the host country’s population. Another negatively associated point is former occasional marijuana smoking. Data from the SAMHSA study showed a noticeable gap between lifetime prevalence and use within the last year [28]. This gap can similarly be observed for cigarette smoking. Our hypothesis is that these differences can be explained by a group of students that tried marijuana consumption for a period of time but then ceased consumption. Thus, a negative correlation can be found in our data. Furthermore, impulsiveness and general anxiety emerged as positive predictors of cigarette smoking. Consistent with our results, numerous previous studies have shown an association among impulsiveness [102], general anxiety [103], and cigarette smoking. The strongest predictors of cigarette smoking were occasional or regular marijuana smoking and risky alcohol consumption. Comparable to our results regarding risky alcohol consumption and marijuana smoking, the group of health behavior variables also had the largest influence on the explained variance in the cigarette smoking regression model. Therefore, there turned out to be a strong association between cigarette smoking and health behavior variables. 

### 4.2. Risky Alcohol Consumption

Regarding potential risk groups for risky alcohol consumption, surprisingly, there was no significant association between gender and risky alcohol consumption in our study, contrary to what has often been reported in the literature [72,104]. However, a few studies have shown results consistent with ours. For example, Santangelo et al. [24,25] reported that 37.9% of female and 40.7% of male students in Palermo, Italy, showed risky alcohol consumption behavior [25]. Significant differences were detected in age and study progress. In particular, younger students and students in their first year of study were significantly more likely to engage in risky alcohol consumption than older students who had been enrolled for longer (>1 year), as also shown by other studies [76].

Sociodemographic factors had a strong influence on risky alcohol consumption in our model. Marginal or part-time employment was associated with risky alcohol consumption. Since socioeconomic status is associated with alcohol consumption, it may be that students who need to earn money in addition to their studies come from families with a lower socioeconomic status [74,105]. Another sociodemographic factor correlated with risky alcohol consumption was migrant background. This factor could have potentially been influenced by students participating in an exchange program [106].

We detected only a small additional influence of psychological (physical and mental health) and psychosocial factors. There were negative associations with loneliness and positive relations with social support from fellow students, procrastination, and impulsiveness. However, these factors explained only a small proportion of the variance in our model. With regard to psychological variables, research results have been heterogeneous. Some previous studies have associated psychological symptoms such as anxiety, loneliness, depression, and illness with increased alcohol consumption [78,80,107,108], although other studies have shown no clear association [11,24,77].

Health behavior variables had the largest impact on risky alcohol consumption in our model. Consistent with previous studies, we observed risky alcohol consumption primarily among students who were physically active [71], heavy social media users [70,75], soft neuroenhancement users [90], and smokers [69]. Elevated scores were measured for occasional and regular cigarette smokers and occasional marijuana smokers. Thus, we can conclude that low health awareness and lifestyle are associated with risky alcohol consumption. When factors were added groupwise, our model was mainly explained by sociodemographic and health behavior variables. Psychological and psychosocial variables only had a weak association with the explained variance in risky consumption, despite the large number of variables tested.

### 4.3. Marijuana Smoking

As with cigarette smoking, female students in our sample were significantly less likely to smoke marijuana than male or diverse students. In addition, our results showed higher rates among students pursuing bachelor’s degrees compared to doctoral candidates and those pursuing the German “Staatsexamen” (studies at university with a final examination administrated by the state—e.g., medicine, pharmacology, law). Regarding the field of study, students of social science, media, and sports smoked marijuana significantly more often than students of STEM, medicine, or education (aspiring teachers). Binary logistic regression analysis revealed that sociodemographic and study-related characteristics such as aspiring to achieve a doctoral degree and having a higher number of semester hours per week were negative predictors of marijuana smoking. In addition to sociodemographic variables, health behavior variables again had the largest impact on marijuana smoking in our model, as we have already seen for cigarette smoking and risky alcohol consumption. Surprisingly, the use of pharmacological and soft neuroenhancements within the previous 12 months was strongly negatively associated with marijuana smoking. Therefore, to avoid confounding with marijuana smoking, the results of our study refer to pharmacological neuroenhancement as excluding smoking marijuana for the purposes of neuroenhancement, as described in the methods section. Positively associated variables included a high AUDIT-C score (indicating riskier alcohol consumption), medium or high physical activity score, and occasional or regular cigarette smoking. Psychological, study-related, and general psychosocial variables had a small impact in our model. In the context of marijuana smoking, the impression emerged that time-consuming, advanced studies (doctoral degrees), as well as trying to improve oneself with neuroenhancement, are not compatible with marijuana smoking. On the other hand, physically active men who also consume more alcohol and cigarettes are clearly more amenable to marijuana. Nevertheless, the use of marijuana as a neuroenhancer should be investigated further.

### 4.4. Limitations

Comparing the groups of students that answered the questionnaire with regard to cigarette smoking, alcohol consumption, and marijuana smoking (3991 students) with the group of students who did not (360 students; exclusion criterion), we found higher rates for male students (+9.8%) and first-year students (+4.3%). Both groups are often associated in the literature with increased rates of risky consumption. Therefore, as a limitation, it should be considered that the prevalence of risky use in our sample may have been underestimated. Tables with full descriptive statistics of excluded participants can be found in the Supplementary Materials.

To measure risky alcohol consumption, we chose the AUDIT-C questionnaire, the short form of the AUDIT questionnaire. We used the short form to keep the entire questionnaire, which already contained about 270 questions, as short as possible. However, the applicability of the AUDIT-C for the detection of risky alcohol consumption is well established [86,87,88]. Furthermore, there are inconsistent data regarding the cut-off of the AUDIT-C to measure risky alcohol consumption. Several studies have presented samples from a clinical setting where a cut-off of ≥3 has been shown to be useful [89,109]. Rumpf et al. [87] showed that in a German nonclinical sample, a cut-off of ≥5 achieved the highest specificity with good sensitivity, which has been confirmed by other studies [88]. Therefore, we decided to use a cut-off of ≥5 for risky alcohol consumption. AUDIT-C scores ≤ 4 were classified as “non-risky consumption”.

To define cigarette smoking and marijuana smoking, we chose to define “current use” as risky, regardless of quantity or frequency (“occasionally” or “regularly”). This decision was based on the works of Inoue-Choi and colleagues [45,46], who were able to show that even occasional cigarette smoking and small amounts of marijuana can be harmful to health. Since 2017, medical marijuana has been approved in Germany for serious or incurable diseases. As a tested medical product, it contains significantly less harmful substances than nonmedical marijuana [110]. Since we did not have any data on medical marijuana in our student collective, we assumed the use of illegal marijuana. This aspect also argues for classifying even small amounts of marijuana as dangerous. Further research on the prevalence of medical cannabis among students would be helpful.

The sample we studied was 70.5% female. Compared to the number of female students enrolled at the Johannes Gutenberg University in Mainz (59.5%) [111], we had an 11% overrepresentation of women in our sample. By adjusting the cut-off values for risky consumption according to gender, we compensated for this limitation as best as possible, but a slight overrepresentation of women must be considered when using our results.

## 5. Conclusions and Practical Recommendations

The present study shows that cigarette smoking, risky alcohol consumption, and marijuana smoking among university students in Germany are associated with multiple factors, especially with health behaviors. Looking more closely at the predictors of the three substances studied, two aspects in particular stand out. First, consumption is predicted mainly by sociodemographic and health behavior variables. Psychological and psychosocial variables play a rather minor role. Second, the consumption behavior of each substance is to a large extent associated with the other two substances we examined. For example, people who drink more alcohol are more likely to smoke cigarettes or marijuana. Those who smoke cigarettes are more likely to consume alcohol or marijuana. This leads us to the point that the consumption of each of the three substances analyzed increases according to the higher risk of consuming the other substances. Conversely, low consumption of one substance is also associated with lower consumption of the other substances (see Table 3, Table 5 and Table 7). This suggests that the substances cannot be considered in isolation from each other, with their consumption being largely interrelated. Based on this association, we conclude that prevention programs could be more effective if they address substance use as a whole and not individual substances (e.g., cigarette smoking, alcohol consumption, marijuana smoking). The second point is that all three substances were very strongly associated with the health behavior variables, as this group had the biggest impact on our regression model. By having one group of variables that have a strong lever to “risky use”, this could also be a good starting point for prevention programs, as it is possible to effectively influence all three substances. For prevention, it might be more a question of “general substance use” than one of specific substance use. This could lead to synergistic effects and make prevention programs more effective.

## Figures and Tables

**Table 1:** Basic and study-related characteristics of the participants.

Variable	Value
**Gender (*n* = 3991)**	
Female	71.4% (*n* = 2848)
Male	27.8% (*n* = 1111)
Diverse	0.8% (*n* = 32)
**Age, range (mean ± SD) (*n* = 3987)**	16–73 (23.8 ± 4.3)
**Semester, range (mean ± SD) (*n* = 3975)**	1–45 (7.2 ± 4.8)
**First year (*n* = 3884)**	
No	83.2% (*n* = 3233)
Yes	16.8% (*n* = 651)
**Aspired degree level (*n* = 3991)**	
Bachelor’s	52.3% (*n* = 2088)
Master’s	21.2% (*n* = 847)
“Staatsexamen”	22.0% (*n* = 878)
Doctoral degree	3.5% (*n* = 139)
Other	1.0% (*n* = 39)
**Field of study (*n* = 3982)**	
STEM	18.1% (*n* = 720)
Social sciences, media, and sports	18.1% (*n* = 719)
Linguistics, humanities, and cultural studies	20.2% (*n* = 804)
Medicine	13.3% (*n* = 530)
Law and economics	12.9% (*n* = 512)
Education (aspiring teachers)	15.5% (*n* = 616)
Other	2.0% (*n* = 81)

**Table 2:** Prevalence of cigarette smoking distributed for the different basic and study-related characteristics.

	Risky Consumption		
Variable	“Yes”	“No”	*p*-Value
**All students (*n* = 3984)**	14.9% (*n* = 592)	85.1% (*n* = 2984)	
**Gender (*n* = 3984)**			
^a^ Female	13.4% (*n* = 381)	86.6% (*n* = 2463)	<0.001 ^a,b; a–c^
^b^ Male	18% (*n* = 200)	82% (*n* = 910)	
^c^ Diverse	34.4% (*n* = 11)	65.6% (*n* = 21)	
**Age, median split (*n*= 3982)**			
^a^ Between 16 and 23	14.5% (*n* = 326)	85.5% (*n* = 1919)	0.486
^b^ Older than 24	15.3% (*n* = 266)	84.7% (*n* = 1471)	
**First year (*n* = 3879)**			
^a^ No	14.8% (*n* = 479)	85.2% (*n* = 2750)	0.516
^b^ Yes	13.8% (*n* = 90)	86.2% (*n* = 560)	
**Aspired degree level (*n* = 3986)**			
^a^ Bachelor’s	16.1% (*n* = 335)	83.9% (*n* = 1752)	0.239
^b^ Master’s	14.1% (*n* = 119)	85.9% (*n* = 726)	
^c^ “Staatsexamen”	13.8% (*n* = 121)	86.2% (*n* = 755)	
^d^ Doctoral degree	8.6% (*n* = 12)	91.4% (*n* = 127)	
^e^ Other	12.8% (*n* = 5)	87.2% (*n* = 34)	
**Field of study (*n* = 3977)**			
^a^ STEM	14.6% (*n* = 105)	85.4% (*n* = 615)	<0.001 ^b–f; c,d; c–f; e,f^
^b^ Social sciences, media, and sports	16.7% (*n* = 120)	83.3% (*n* = 598)	
^c^ Linguistics, humanities, and cultural sciences	18.3% (*n* = 147)	81.7% (*n* = 656)	
^d^ Medicine	11.2% (*n* = 59)	88.8% (*n* = 469)	
^e^ Law and economics	16.4% (*n* = 84)	83.6% (*n* = 428)	
^f^ Education (aspiring teachers)	9.8% (*n* = 60)	90.2% (*n* = 555)	
^g^ Other	18.5% (*n* = 15)	81.5% (*n* = 66)	

*p*-value provided by Pearson’s chi-square test. ^a–g^ superscript letter pairs indicate significant differences between variable categories.

**Table 3:** Odds ratios (OR) and 95% confidence intervals (CI) for the dependent variable smoking cigarettes and each predictor variable (*p* ≤ 0.05).

Variable	OR (95% CI)	*p*-Value	Wald Chi-Square
Migrant background (yes)	0.585 (0.444–0.772)	<0.001	14.354
Use of soft neuroenhancement (within the last 12 months)	0.593 (0.428–0.823)	0.002	9.795
Marijuana consumption (formerly occasionally)	0.790 (0.629–0.990)	0.041	4.178
Impulsiveness	1.106 (1.049–1.166)	<0.001	13.797
Anxiety symptoms	1.114 (1.014–1.224)	0.028	5.066
AUDIT-C-score	1.336 (1.262–1.415)	<0.001	99.144
Marijuana consumption (currently occasionally)	1.488 (1.153–1.919)	0.002	9.354
First study (did other studies before, but did not graduate)	2.413 (1.099–5.298)	0.028	4.817
Marijuana consumption (currently regularly)	2.971 (1.896–4.655)	<0.001	22.591

**Table 4:** Prevalence of risky alcohol consumption (AUDIT-C score: female ≥4, male ≥5) distributed for the different basic and study-related characteristics (*n* = 3984).

	Risky Consumption		
Variable	“Yes”	“No”	*p*-Value
**All Students (*n* = 3984)**	38.6% (*n* = 1537)	61.4% (*n* = 2447)	
**Gender (*n* = 3984)**			
^a^ Female	37.9% (*n* = 1076)	62.1% (*n* = 1766)	0.120
^b^ Male	40.7% (*n* = 452)	59.3% (*n* = 658)	
^c^ Diverse	28.1% (*n* = 9)	71.9% (*n* = 23)	
**Age, median split (*n* = 3981)**			
^a^ Between 16 and 23	40.8% (*n* = 917)	59.2% (*n* = 1328)	<0.001 ^a,b^
^b^ Older than 24	35.7% (*n* = 620)	64.3% (*n* = 1116)	
**First Year (*n* = 3878)**			
^a^ No	37.8% (*n* = 1219)	62.2% (*n* = 2009)	0.030 ^a,b^
^b^ Yes	42.3% (*n* = 275)	57.7% (*n* = 375)	
**Aspired degree level (*n* = 3984)**			
^a^ Bachelor’s	40% (*n* = 835)	60% (*n* = 1251)	0.143
^b^ Master’s	36.7% (*n* = 310)	63.3% (*n* = 535)	
^c^ “Staatsexamen”	38.7% (*n* = 339)	61.3% (*n* = 537)	
^d^ Doctoral degree	27.3% (*n* = 38)	72.7% (*n* = 101)	
^e^ Other	39.5% (*n* = 15)	60.5% (*n* = 23)	
**Field of Study (*n* = 3975)**			
^a^ STEM	36.4% (*n* = 262)	63.6% (*n* = 457)	0.001 ^b,c; c–e^
^b^ Social sciences, media, and sports	43.2% (*n* = 310)	56.8% (*n* = 407)	
^c^ Linguistics, humanities, and cultural sciences	33.5% (*n* = 269)	66.5% (*n* = 534)	
^d^ Medicine	37.5% (*n* = 198)	62.5% (*n* = 330)	
^e^ Law and economics	43% (*n* = 220)	57% (*n* = 292)	
^f^ Education (aspiring teachers)	38.6% (*n* = 238)	61.4% (*n* = 378)	
^g^ Other	46.3% (*n* = 37)	53.8% (*n* = 43)	

*p*-value provided by Pearson’s chi-square test. ^a–g^ superscript letter pairs indicate significant differences between variable categories.

**Table 5:** Odds ratios (OR) and 95% confidence intervals (CI) for the dependent variable risky alcohol consumption (AUDIT-C score: female ≥4, male ≥5) and each predictor variable (*p* ≤ 0.05).

Variable	OR (95% CI)	*p*-Value	Wald Chi-Square
Use of pharmacological neuroenhancement (within the last 12 months)	0.625 (0.423–0.924)	0.019	5.542
First study (did other studies before, but changed the field of study)	0.693 (0.549–0.875)	0.002	9.525
Relationship status (joint household)	0.736 (0.580–0.933)	0.011	6.420
First study (no, graduated before)	0.745 (0.563–0.985)	0.039	4.276
Use of soft neuroenhancement (within the last 12 months)	0.799 (0.647–0.988)	0.038	4.287
Fruit consumption	0.822 (0.753–0.898)	<0.001	18.905
Loneliness	0.931 (0.892–0.972)	0.001	10.749
Procrastination	1.017 (1.001–1.032)	0.038	4.311
Impulsiveness	1.100 (1.052–1.150)	<0.001	17.436
Social support by students	1.205 (1.079–1.345)	0.001	10.989
WHO scale for physical activity (active, beneficial to health)	1.303 (1.053–1.613)	0.015	5.915
Use of social media	1.323 (1.242–1.411)	<0.001	74.190
Employment (yes, marginally employed)	1.381 (1.137–1.678)	0.001	10.595
Cigarette smoking (currently regularly)	1.426 (1.030–1.974)	0.033	4.570
Migrant background (yes)	1.523 (1.226–1.893)	<0.001	14.394
Employment (yes, part-time)	1.527 (1.164–2.001)	0.002	9.374
Marijuana consumption (currently occasionally)	1.600 (1.194–2.143)	0.002	9.908
Cigarette smoking (currently occasionally)	1.691 (1.297–2.203)	<0.001	15.113

**Table 6:** Prevalence of marijuana smoking distributed for the different basic and study-related characteristics (*n* = 3985).

	Risky Consumption		
Variable	“Yes”	“No”	*p*-Value
**All students (*n* = 3985)**	10.9% (*n* = 435)	89.1% (*n* = 3550)	
**Gender (*n* = 3985)**			
^a^ Female	9.0% (*n* = 256)	91.0% (*n* = 2587)	<0.001 ^a,b; a–c^
^b^ Male	15.4% (*n* = 171)	84.6% (*n* = 939)	
^c^ Diverse	25.0% (*n* = 8)	75.0% (*n* = 24)	
**Age, median split (*n* = 3981)**			
^a^ Between 16 and 23	11.8% (*n* = 266)	88.2% (*n* = 1979)	0.034 ^a,b^
^b^ Older than 24	9.7% (*n* = 169)	90.3% (*n* = 1567)	
**First year (*n* = 3878)**			
^a^ No	10.8% (*n* = 348)	89.2% (*n* = 2881)	0.725
^b^ Yes	11.2% (*n* = 73)	88.8% (*n* = 576)	
**Aspired degree level (*n* = 3985)**			
^a^ Bachelor’s	13.0% (*n* = 272)	87.0% (*n* = 1815)	<0.001 ^a–c; a–d^
^b^ Master’s	9.6% (*n* = 81)	90.4% (*n* = 764)	
^c^ “Staatsexamen”	8.7% (*n* = 76)	91.3% (*n* = 800)	
^d^ Doctoral degree	3.6% (*n* = 5)	96.4% (*n* = 134)	
^e^ Other	2.6% (*n* = 1)	97.4% (*n* = 37)	
**Field of study (*n* = 3977)**			
^a^ STEM	9.3% (*n* = 67)	90.7% (*n* = 653)	<0.001 ^a,b; b–d; b–f^
^b^ Social sciences, media, and sports	15.7% (*n* = 113)	84.3% (*n* = 605)	
^c^ Linguistics, humanities, and cultural sciences	10.8% (*n* = 87)	89.2% (*n* = 716)	
^d^ Medicine	8.3% (*n* = 44)	91.7% (*n* = 484)	
^e^ Law and economics	10.7% (*n* = 55)	89.3% (*n* = 457)	
^f^ Education (aspiring teachers)	9.1% (*n* = 56)	90.9% (*n* = 559)	
^g^ Other	16.0% (*n* = 13)	84.0% (*n* = 68)	

*p*-value provided by Pearson’s chi-square test. ^a–g^ superscript letter pairs indicate significant differences between variable categories.

**Table 7:** Odds ratios (OR) and 95% confidence intervals (CI) for the dependent variable smoking marijuana and each independent variable (*p* ≤ 0.05).

Variable	OR (95% CI)	*p*-Value	Wald Chi-Square
Aspired degree (Ph.D.)	0.169 (0.038–0.744)	0.019	5.452
Use of pharmacological neuroenhancement (within the last 12 months)	0.646 (0.437–0.954)	0.028	4.861
Use of soft neuroenhancement (within the last 12 months)	0.691 (0.488–0.978)	0.037	4.329
Self-harm	0.827 (0.699–0.978)	0.027	5.117
Semester hours per week	0.977 (0.963–0.990)	0.001	11.023
AUDIT-C score	1.209 (1.140–1.282)	<0.001	39.911
Gender (male)	1.314 (1.008–1.713)	0.043	4.466
First study (did other studies before, but changed the field of study)	1.398 (1.052–1.858)	0.021	4.910
WHO scale for physical activity (moderate active)	1.451 (1.008–2.089)	0.045	3.967
WHO scale for physical activity (active, beneficial to health)	1.641 (1.200–2.245)	0.002	9.411
Cigarette smoking (currently regularly)	1.959 (1.476–2.599)	<0.001	30.126
Cigarette smoking (currently occasionally)	2.007 (1.568–2.568)	<0.001	21.728

## Data Availability

The raw data supporting the conclusions of this article will be made available by the corresponding author, without undue reservations.

## Supplementary Materials

The following supporting information can be downloaded at: https://www.mdpi.com/article/10.3390/healthcare11243182/s1, Table S1: List of all 56 variables used in the present study and their characteristics; Table S2: Association between the dependent variable cigarette smoking and each independent variable (pretest for regression); Table S3: Association between the dependent variable risky alcohol consumption and each independent variable (pretest for regression); Table S4: Association between the dependent variable marijuana smoking and each independent variable (pretest for regression); Table S5: Answering rate for each independent variable (included group, *N* = 3991); Table S6: Answering rate for each independent variable (non-included group, *N* = 360) [85,93,112,113,114,115,116,117,118,119,120,121,122,123,124,125,126,127,128,129,130,131,132,133,134,135,136].
