# Supplementary material for: Cigarette Smoking, Risky Alcohol Consumption, and Marijuana Smoking among University Students in Germany: Identification of Potential Sociodemographic and Study-Related Risk Groups and Predictors of Consumption
Source: Healthcare (Basel). 2023 Dec 16;11(24):3182. doi: 10.3390/healthcare11243182 (PMC10742791; doi:10.3390/healthcare11243182)
Supplement: Supplementary file 1 [file healthcare-11-03182-s001.zip › healthcare-2686482-supplementary.pdf]

healthcare

MDPI

Supplementary Materials

Cigarette Smoking, Risky Alcohol Consumption, and Marijuana Smoking among University Students in Germany: Identification of Potential Sociodemographic and Study-Related Risk Groups and Predictors of Consumption

Thilo A. Hoff [1], Sebastian Heller [1], Jennifer L. Reichel [1], Antonia M. Werner [2], Markus Schäfer [3,†], Ana Nanette Tibubos [2,4], Perikles Simon [5], Manfred E. Beutel [2], Stephan Letzel [1], Thomas Rigotti [6,7] and Pavel Dietz [1,*]

1 Institute of Occupational, Social and Environmental Medicine, University Medical Centre of the Johannes Gutenberg University, 55131 Mainz, Germany; thilo.hoff@web.de (T.A.H.); seheller@uni-mainz.de (S.H.); jreichel@uni-mainz.de (J.L.R.); letzel@uni-mainz.de (S.L.)
2 Department of Psychosomatic Medicine and Psychotherapy, University Medical Center of the Johannes Gutenberg University, 55131 Mainz, Germany; antonia.werner@unimedizin-mainz.de (A.M.W.); tibubos@uni-trier.de (A.N.T.); manfred.beutel@unimedizin-mainz.de (M.E.B.)
3 Department of Communication, Johannes Gutenberg University, 55122 Mainz, Germany; markus.schaefer@uni-mainz.de
4 Nursing Science, Diagnostics in Healthcare and E-Health, Trier University, 54296 Trier, Germany
5 Department of Sports Medicine, Rehabilitation and Disease Prevention, Institute of Sport Science, Johannes Gutenberg University, 55122 Mainz, Germany; simonpe@uni-mainz.de
6 Department of Work, Organizational and Business Psychology, Institute of Psychology, Johannes Gutenberg University, 55122 Mainz, Germany; rigotti@uni-mainz.de
7 Leibniz Institute of Resilience Research, 55122 Mainz, Germany
* Correspondence: pdietz@uni-mainz.de; Tel.: +49-6131-17-9213
† Current address: Department of Media and Communication Management, Macromedia University of Applied Sciences, 60488 Frankfurt, Germany.

Supplementary Table S1. List of all 56 variables used in the present study and their characteristics.

Dimension	Variable (count)	Scale	Reference	Item	"Question for the self-constructed item," *answering options*
Sociodemographic variables	Gender (1)	Self-constructed items		1	"Your gender": *male (1) / female (2) / diverse (3)*
	Age (1)	Self-constructed items		1	"Your age in years"
	Relationship status (1)	Self-constructed items		1	"What is your relationship status?" *Single (1) / Partnership in one household*

			(2) / Partnership with separate living conditions (3)
Employment (1)	Self-constructed items	1	"Are you currently employed?" *No (1) / Yes, full-time (2) / Yes, regularly part-time (3) / Yes, marginally or irregularly employed (4)*
Financial situation	Self-constructed items	1	"How much money do you have at your disposal on average each month?"
Parental support	Self-constructed items	1	"How do you finance your studies?" *Support by parents or relatives: Yes / No*
Parents' educational background	Self-constructed items	1	"Please select the highest educational attainment of your mother or your father (the highest of both)": *List of attainments, also including "other attainment"*
Migrant background	Self-constructed items	1	"Were both of your parents born within the current geography of Germany?" *Yes (1) / No (2) / Don't know (3) / No information (4)*
Personal migration experience (1)	Self-constructed items	1	"Were you born in the current geography of Germany?" *Yes (1) / No (2) / Don't know (3) / No information (4)*
Degree (1)	Self-constructed items	1	"Which degree are you pursuing in your current studies?" *Drop-down list of all degrees at the University of Mainz*
Field of study (1)	Self-constructed items	1	Grouped variable created for "What is your current study subject or special subject?" *Drop-down list of all subjects of the University of Mainz*
First year (Semester / study progress) (1)	Self-constructed items	1	"What is the number of your current university semester?"

	First study	Self-constructed items		1	"Have you started or completed another degree program before your current one?" *No (1) / Yes, graduate before (2) / Yes, but changed the field of study (3) / Yes, but did not graduate (4)*
	Semester hours and semester hours with mandatory attendance (2)	Self-constructed items		2	"How many hours per week are you taking this semester?" and "Of these, how many require your attendance?"
Psychological variables	Somatic symptoms	SSS8	Gierk et al. (2014) [120]	8	
	Depression symptoms	PHQ-9	Kroenke et al. (2001) [126]	9	
	General anxiety	GAS-2	Döring & Bortz (1993) [114]	2	
	Social anxiety	Mini-SPIN	Wiltink et al. (2017) [136]	3	
	Loneliness	UCLA	Döring & Bortz (1993) [114]	3	
	Emotional exhaustion	MBI-SS	Gumz et al. (2013) [121]	5	
Study-related psychosocial variables	Presenteeism		Töpritz et al. (2016) [134]	1	
	Absenteeism		Töpritz et al. (2016) [134]	1	
	Perceived employability	Self-constructed items		1	"How do you estimate your chances on the job market?" Very Bad (1) to Very Good (7)

Autonomy	BARI-S	Gusy & Lohmann (2014) [122]	6
Coping through the use of structural resources and social support (2)		Daniels et al. (2008) [112]	4
Self-endangering behavior		Krause et al. (2015) [125]	
Planning competency		Dettmers & Clauß (2018) [113]	8
Work complexity		Gusy et al. (2016) [123]	
Competition		Fend (1997) [119]	4
Performance pressure		Fend (1997) [119]	5
Cognitive irritation		Mohr et al. (2005) [127]	
Social support by teachers	SALSA	Rimann & Udris (1997) [129]	3
Social support by fellow students	SALSA	Rimann & Udris (1997) [129]	3
Competence for self-motivation		Dettmers & Clauß (2018) [113]	8

	Self-efficacy		Self-Efficacy Scale, adapted from Rigotti et al. (2008) [128]	6	
	Procrastination	GPS-K	Klingsieck & Fries (2012) [128]	9	
General psychosocial variables	Self-criticism	DEQ-SC4	Rudich et al. (2008) [130]	4	
	Self-acceptance		Ryff & Keyes (1995); Ryff (1989) [131-132]	3	
	Emotion regulation	ERQ	Abler & Kessler (2011) [115]	4	
	Emotion suppression	ERQ	Abler & Kessler (2011) [115]	4	
	Impulsiveness	ERQ	Abler & Kessler (2011) [115]	4	
Health behavior variables	Healthy diet	Self-constructed items		1	"A healthy diet consists of eating a large quantity of whole grains, potatoes, fruit, and vegetables several times a day and food low in trans fats as well as a small quantity of red meat and sausages, fast-food (e.g., burger, fries), and sugar-rich food (e.g., lemonade, soft drinks, candy, cake). To what extend have you study to a healthy diet in the last month?" Not

				at all (1) to Very strong (5)
Fruit consumption		European Comission (2013); Fehr et al. (2018) [117-118]	4	
Vegetable consumption		European Comission (2013); Fehr et al. (2018) [117-118]	4	
Physical activity	IPAQ Short version	Craig et al. (2003); International Physical Activity Questionnaire [116, 135]		
Sedentary behavior	IPAQ Short version	Craig et al. (2003); International Physical Activity Questionnaire [116, 135]	7	
Smoking cigarettes		Töpritz et al. (2016) [134]	1	
Risky alcohol consumption	AUDIT-C	Bush et al. (1998) [85]	1	
Marijuana smoking	Self-constructed items			"Do you smoke marijuana?" *never (1) / formerly occasionally (2) / formerly regularly (3) / currently occasionally (4) / currently regularly (5)*

Use of neuroenhancement (2)	Dietz et al. (2016) [93]	2
Use of social-media	Adapted from Stark et al. (2017) [133]	6

Supplementary Table S2. Association between the dependent variable cigarette smoking and each independent variable (pre-test for regression).

Variable	*p*-value
**1) Sociodemographic variables**	
Gender	[c] <.001
Age, median split	[b] .486
Relationship status	[c] .430
Employment	[c] <.001
Financial situation	[a] <.001
Parental support	[c] .159
Parents' educational background	[c] .278
Migrant background	[c] <.001
Personal migrant experience	[c] .001
Aspired degree	[c] .095
Field of study	[c] <.001
Semester	[a] .921
First study	[c] .001

First year	[c] .516
Semester hours per week	[a] .005
Semester hours per week with mandatory attendance	[a] .001
**2) Psychological variables**	
Somatic symptoms	[a] <.001
Depression symptoms	[a] <.001
General anxiety	[a] <.001
Social anxiety	[a] .663
Loneliness	[a] .374
Emotional exhaustion	[a] .002
**3) Study-related psychosocial variables**	
Presenteeism	[a] .428
Absenteeism	[a] .001
Perceived employability	[a] .002
Autonomy	[a] .990
Autonomy exams	[a] .123
Coping via use of structural resources	[a] .188
Coping via use of social resources	[a] .198
Self-endangering behavior	[a] .813
Planning competency	[a] <.001

Work complexity	[a] .603
Competition	[a] .421
Performance pressure	[a] .064
Cognitive irritation	[a] .064
Social support from teachers	[a] .294
Social support from fellow students	[a] .401
Competence for self-motivation	[a] <.001
Self-efficacy	[a] .001
Procrastination	[a] <.001
**4) General psychosocial variables**	
Self-criticism	[a] .059
Self-acceptance	[a] <.001
Emotion regulation	[a] .209
Emotion suppression	[a] .316
Impulsiveness	[a] <.001
**5) Health behavior variables**	
Healthy diet	[b] <.001
Fruit consumption	[a] .010
Vegetable consumption	[a] .377
Physical activity	[b] .347

Sedentary behavior	[a] .101
AUDIT-C score	[a] <.001
Marijuana smoking	[c] <.001
Use of soft neuroenhancement	[c] <.001
Use of pharmacological neuroenhancement	[c] <.001
Use of social media	[a] <.001

[a] ANOVA test.
[b] Pearson's chi-square test.
[c] Cramer's V-test.

Supplementary Table S3. Association between the dependent variable risky alcohol consumption and each independent variable (pretest for regression).

**Variable**	***p*-value**
**1) Sociodemographic variables**	
Gender	[c] .120
Age, median split	[b] .001
Relationship status	[c] <.001
Employment	[c] <.001
Financial situation	[a] .007
Parental support	[c] <.001
Parents' educational background	[c] .118
Migrant background	[c] <.001

Personal migrant experience	[c] .002
Aspired degree	[c] .032
Field of study	[c] .001
Semester	[a] <.001
First study	[c] .001
First year	[c] .030
Semester hours per week	[a] .418
Semester hours per week with mandatory attendance	[a] .206
**2) Psychological variables**	
Somatic symptoms	[a] .767
Depression symptoms	[a] .339
General anxiety	[a] <.001
Social anxiety	[a] <.001
Loneliness	[a] <.001
Emotional exhaustion	[a] .089
**3) Study-related psychosocial variables**	
Presenteeism	[a] .020
Absenteeism	[a] .616
Perceived employability	[a] .839
Autonomy	[a] .318

Autonomy exams	[a] .608
Coping via use of structural resources	[a] .372
Coping via use of social resources	[a] .949
Self-endangering behavior	[a] .011
Planning competency	[a] .432
Work complexity	[a] .930
Competition	[a] .089
Performance pressure	[a] .010
Cognitive irritation	[a] <.001
Social support by teachers	[a] .590
Social support by fellow students	[a] <.001
Competence for self-motivation	[a] .447
Self-efficacy	[a] .149
Procrastination	[a] <.001
**4) General psychosocial variables**	
Self-criticism	[a] .239
Self-acceptance	[a] <.001
Emotion regulation	[a] .762
Emotion suppression	[a] <.001
Impulsiveness	[a] <.001

5) Health behavior variables

Healthy diet	[b] .004
Fruit consumption	[a] <.001
Vegetable consumption	[a] .326
Physical activity	[b] <.001
Sedentary behavior	[a] .006
Cigarette smoking	[c] <.001
Marijuana smoking	[c] <.001
Use of soft neuroenhancement	[c] <.001
Use of pharmacological neuroenhancement	[c] <.001
Use of social media	[a] <.001

[a] ANOVA test.

[b] Pearson's chi-square test.

[c] Cramer's V-test.

Supplemental Table S4. Association between the dependent variable marijuana smoking and each independent variable (pretest for regression)

Variable	*p*-value
**1) Sociodemographic variables**	
Gender	[c] <.001
Age, median split	[b] .034
Relationship status	[c] .014
Employment	[c] .012
Finances	[a] .193
Parental support	[c] .028
Parents' education	[c] .189
Parents' migration	[c] .479
Own migration	[c] .727

Aspired degree	[c] <.001
Field of study	[c] <.001
Semester	[a] .028
First study	[c] <.001
First year	[c] .725
Semester hours per week	[a] <.001
Semester hours per week in attendance	[a] .007
**2) Psychological variables**	
Physical complaints	[a] .380
Depression symptoms	[a] <.001
Anxiety symptoms	[a] .355
Social anxiety	[a] .535
Loneliness	[a] .235
Emotional exhaustion	[a] .851
**3) Study-related psychosocial variables**	
Presenteeism	[a] .070
Absenteeism	[a] .279
Perceived employability	[a] .659
Autonomy	[a] .231
Autonomy exams	[a] .650
Coping via use of structural resources	[a] .297
Coping via use of social resources	[a] .239
Self-endangering behavior	[a] <.001
Planning competency	[a] <.001
Work complexity	[a] .249
Competition	[a] .335
Performance pressure	[a] <.001
Cognitive irritation	[a] .201
Social support from teachers	[a] .423
Social support from fellow students	[a] .906
Competence for self-motivation	[a] <.001
Self-efficacy	[a] .401
Procrastination	[a] <.001
**4) General psychosocial variables**	
Self-criticism	[a] .541
Self-acceptance	[a] .279
Emotion regulation	[a] .365
Emotion suppression	[a] .867
Impulsiveness	[a] .002
**5) Health behavior variables**	
Healthy diet	[b] .249
Fruit consumption	[a] .990

Vegetable consumption	[a] .241
Physical activity	[b] <.001
Sedentary behavior	[a] .091
AUDIT-C score	[a] <.001
Cigarette smoking	[c] <.001
Use of soft neuroenhancement	[c] <.001
Use of pharmacological Neuroenhancement	[c] <.001
Use of social media	[a] .011

a ANOVA test.
b Pearson's chi-square test.
c Cramer's V-test.

Supplemental Table S5. Answering rate for each independent variable (included group, *N* = 3991)

**Variable**	*Answering*	**Percentage**
**1) Sociodemographic variables**		
Gender	3991	100.0%
Age	3987	99.9%
Relationship status	3987	99.9%
Employment	3991	100.0%
Financial situation	3911	98.0%
Parental support	3968	99.4%
Parents' educational background	3989	99.9%
Migrant background	3990	100.0%
Personal migration experience	3990	100.0%
Aspired degree	3991	100.0%
Field of study	3982	99.8%
Semester	3975	99.6%
First study	3989	99.9%
First year	3884	97.3%
Semester hours per week	3867	96.9%
Semester hours per week with mandatory attendance	3873	97.0%
**2) Psychological variables**		
Physical complaints	3988	99.9%
Depression symptoms	3987	99.9%
Anxiety symptoms	3990	100.0%
Social anxiety	3617	90.6%

Loneliness	3991	100.0%
Emotional exhaustion	3909	97.9%
**3) Study-related psychosocial variables**		
Presenteeism	3897	97.6%
Absenteeism	3898	97.7%
Perceived employability	3892	97.5%
Autonomy	3949	98.9%
Autonomy exams	3943	98.8%
Coping via use of structural resources	3937	98.6%
Coping via use of social resources	3941	98.7%
Self-endangering behavior	3935	98.6%
Planning competency	3935	98.6%
Work complexity	3922	98.3%
Competition	3921	98.2%
Performance pressure	3920	98.2%
Cognitive irritation	3909	97.9%
Social support by teachers	3892	97.5%
Social support by fellow students	3900	97.7%
Competence for self-motivation	3891	97.5%
Self-efficacy	3890	97.5%
Procrastination	3932	98.5%
**4) General psychosocial variables**		
Self-criticism	3881	97.2%
Self-acceptance	3880	97.2%
Emotion regulation	3879	97.2%
Emotion suppression	3880	97.2%
Impulsiveness	3874	97.1%
**5) Health behavior variables**		
Healthy diet	3991	100.0%
Fruit consumption	3649	91.4%
Vegetable consumption	3806	95.4%
Physical activity	3899	97.7%
Sedentary behavior	3899	97.7%
Use of soft neuroenhancement	3984	99.8%
Use of pharmacological neuroenhancement	3984	99.8%
Use of social media	3987	99.9%
Average	3923	98.3%

Supplemental Table S6. Answering rate for each independent variable (non-included group, *N* = 360)

Variable	*answering*	per-cent
**1) Sociodemographic variables**		
Gender	359	99.7%
Age	360	100.0%
Relationship status	360	100.0%
Employment	95	26.4%
Financial situation	0	0.0%
Parental support	0	0.0%
Parents' educational background	360	100.0%
Migrant background	360	100.0%
Personal migration experience	360	100.0%
Aspired degree	360	100.0%
Field of study	360	100.0%
Semester	358	99.4%
First study	360	100.0%
First year	342	95.0%
Semester hours per week	0	0.0%
Semester hours per week with mandatory attendance	0	0.0%
**2) Psychological variables**		
Physical complaints	340	94.4%
Depression symptoms	320	88.9%
Anxiety symptoms	297	82.5%
Social anxiety	267	74.2%
Loneliness	297	82.5%
Emotional exhaustion	0	0.0%
**3) Study-related psychosocial variables**		
Presenteeism	0	0.0%
Absenteeism	0	0.0%
Perceived employability	0	0.0%
Autonomy	0	0.0%
Autonomy exams	0	0.0%
Coping via use of structural resources	0	0.0%
Coping via use of social resources	0	0.0%
Self-endangering behavior	0	0.0%
Planning competency	0	0.0%
Work complexity	0	0.0%
Competition	0	0.0%
Performance pressure	0	0.0%
Cognitive irritation	0	0.0%
Social support by teachers	0	0.0%
Social support by fellow students	0	0.0%

Competence for self-motivation	0	0.0%
Self-efficacy	0	0.0%
Procrastination	0	0.0%
**4) General psychosocial variables**		
Self-criticism	0	0.0%
Self-acceptance	0	0.0%
Emotion regulation	0	0.0%
Emotion suppression	0	0.0%
Impulsiveness	0	0.0%
**5) Health behavior variables**		
Healthy diet	153	42.5%
Fruit consumption	124	34.4%
Vegetable consumption	100	27.8%
Physical activity	29	8.1%
Sedentary behavior	29	8.1%
Use of soft neuroenhancement	0	0.0%
Use of pharmacological neuroenhancement	0	0.0%
Use of social media	224	62.2%
Average	117	32.6%
